# Complement-mediated serum bactericidal activity of antibodies elicited by the *Shigella sonnei* GMMA vaccine in adults from a shigellosis-endemic country: Exploratory analysis of a Phase 2a randomized study

**DOI:** 10.3389/fimmu.2022.971866

**Published:** 2022-09-09

**Authors:** Melissa C. Kapulu, Usman Nakakana, Antonella S. Sciré, Eleanna Sarakinou, Valentino Conti, Omar Rossi, Alessandra Acquaviva, Francesca Necchi, Christina W. Obiero, Laura B. Martin, Philip Bejon, Patricia Njuguna, Francesca Micoli, Audino Podda

**Affiliations:** ^1^ Kenya Medical Research Institute (KEMRI)-Wellcome Trust Programme, Kilifi, Kenya; ^2^ Centre for Tropical Medicine and Global Health, Nuffield Department of Medicine, University of Oxford, Oxford, United Kingdom; ^3^ GSK Vaccines Institute for Global Health, Siena, Italy; ^4^ Clinical Research Department, KEMRI-Wellcome Trust Programme, Kilifi, Kenya

**Keywords:** *shigella sonnei*, generalized modules for membrane antigens (GMMA), 1790GAHB vaccine, functional antibody, serum bactericidal activity, IgG, kenyan adults

## Abstract

*Shigella* is associated with a significant burden of disease worldwide among individuals of all ages and is the major cause of moderate and severe diarrhea in children under five years of age in low- and middle-income countries. Several candidate vaccines against *Shigella* species are currently under clinical development. The investigational 1790GAHB vaccine against *Shigella sonnei* is based on GMMA (Generalized Modules for Membrane Antigens) technology. The vaccine was well tolerated and induced high antibody levels in early-phase clinical trials in both *Shigella*-endemic and non-endemic settings. The present analysis assessed the bactericidal activity of antibodies induced by 1790GAHB in healthy Kenyan adults during a phase 2a, controlled, randomized study (NCT02676895). Participants received two doses of 1790GAHB 4 weeks apart containing either 1.5/25 µg or 6/100 µg O antigen/protein, or active comparator vaccines (Control). Serum bactericidal activity (SBA) against *S. sonnei* was assessed at pre-vaccination (D1), 28 days post-first dose (D29) and 28 days post-second dose (D57), using a luminescence-based assay. Most participants had SBA titers above the lower limit of quantification of the assay at D1. SBA geometric mean titers increased 3.4-fold in the 1.5/25 µg group and 6.3-fold in the 6/100 µg group by D29 and were maintained at D57. There was no increase in SBA geometric mean titers in the Control group. A strong correlation was observed between SBA titers and anti-*S. sonnei* lipopolysaccharide serum immunoglobulin G antibody concentrations (Pearson correlation coefficient = 0.918), indicating that SBA can effectively complement enzyme-linked immunosorbent assay data by indicating the functionality of 1790GAHB-induced antibodies.

## Graphical Abstract

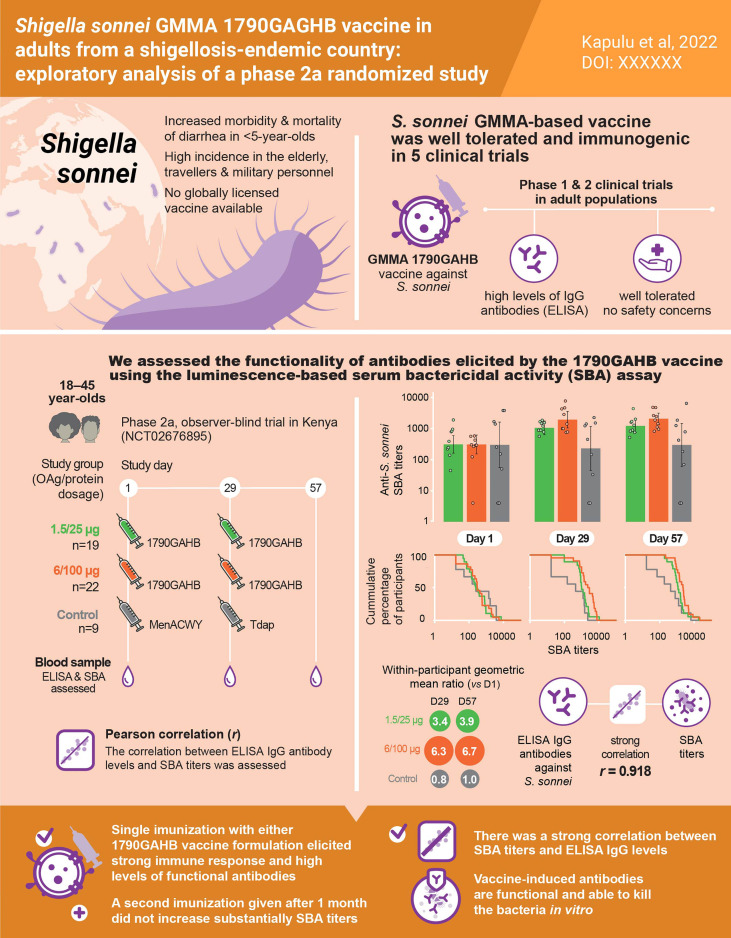

## Introduction

*Shigella* is a leading global cause of diarrheal disease and diarrheal-related mortality (1–3). It is especially responsible for causing diarrhea in children under the age of 5 years in developing countries (4, 5). In the Global Enteric Multicentre Study (GEMS) conducted in Africa and South Asia, *Shigella* infection was shown to be the third, second, and first cause of moderate to severe diarrhea in children aged 0–11 months, 12–23 months, and 24–59 months respectively (4). *Shigella* also has high incidence in adults, in particular older ones, and travelers and military personnel (3, 6, 7). The true burden of *Shigella* is likely unknown, especially in low- and middle-income countries, where access to molecular detection methods is limited. In the GEMS study and its 1-year follow-up, most children with quantitative polymerase chain reaction-confirmed *Shigella* were culture negative (8, 9), indicating a substantial underestimation of the true burden of disease in shigellosis-endemic settings.

In target populations, treatment options for shigellosis are limited and may include appropriate antibiotic use. However, antibiotic resistance is increasing and many *Shigella* isolates are resistant to common antibiotics (10–12). The Centers for Disease Control and Prevention’s 2019 Antibiotic Resistance Threats Report classified drug-resistant *Shigella* as a serious threat, with an estimated 77,000 infections per year (13). Fluoroquinolone-resistant *Shigella* species has received medium priority from the World Health Organization for research and development of new antibiotics (14). In this context, the development of an effective vaccine against *Shigella* is even more important.

The genus *Shigella* comprises four species (*S. flexneri*, *S. sonnei*, *S. dysenteriae*, and *S. boydii*) with more than 50 serotypes that are differentiated based on the structure of the lipopolysaccharide (LPS) O antigen (OAg) anchored in the outer membrane of the bacteria (15). OAg is considered the dominant protective antigen and is the main target of vaccine development strategies (16, 17). Several mono- or multicomponent candidate vaccines against *Shigella* species are currently under development (18–24) to address this unmet medical need. The investigational vaccine against *S. sonnei* (1790GAHB) developed using the Generalized Modules for Membrane Antigens (GMMA) technology was demonstrated to be well tolerated and immunogenic in healthy adults from both *Shigella* endemic and non-endemic regions (25–27). GMMA are highly immunogenic genetically modified bacterial outer membrane particles used as antigen delivery system for OAg (28–30).

The predominant readout for the immunogenicity of *S. sonnei* 1790GAHB vaccine has been serum immunoglobulin G (IgG) antibody level against LPS OAg. Serum bactericidal activity (SBA) has been detected in infected individuals living in *Shigella*-endemic regions (31, 32). Even if an immunological correlate of protection is not yet established for *Shigella*, functional antibodies might be a relevant indicator of immune protection (33). We recently reported the bactericidal activity of sera collected from adults who received 3 doses of 1790GAHB during a phase 1 trial in France (34). Some of these participants also received a booster dose. The analysis showed that vaccination induced functional serum antibodies, active against *S. sonnei* in bactericidal assays. The booster dose administered 2–3 years following the primary schedule induced a strong increase in SBA titers in most of the previously vaccinated participants. The study also demonstrated high correlations between human anti-*S. sonnei* LPS IgG antibodies elicited by 1790GAHB and their functionality in terms of SBA against *S. sonnei* (34).

**Figure 1:**
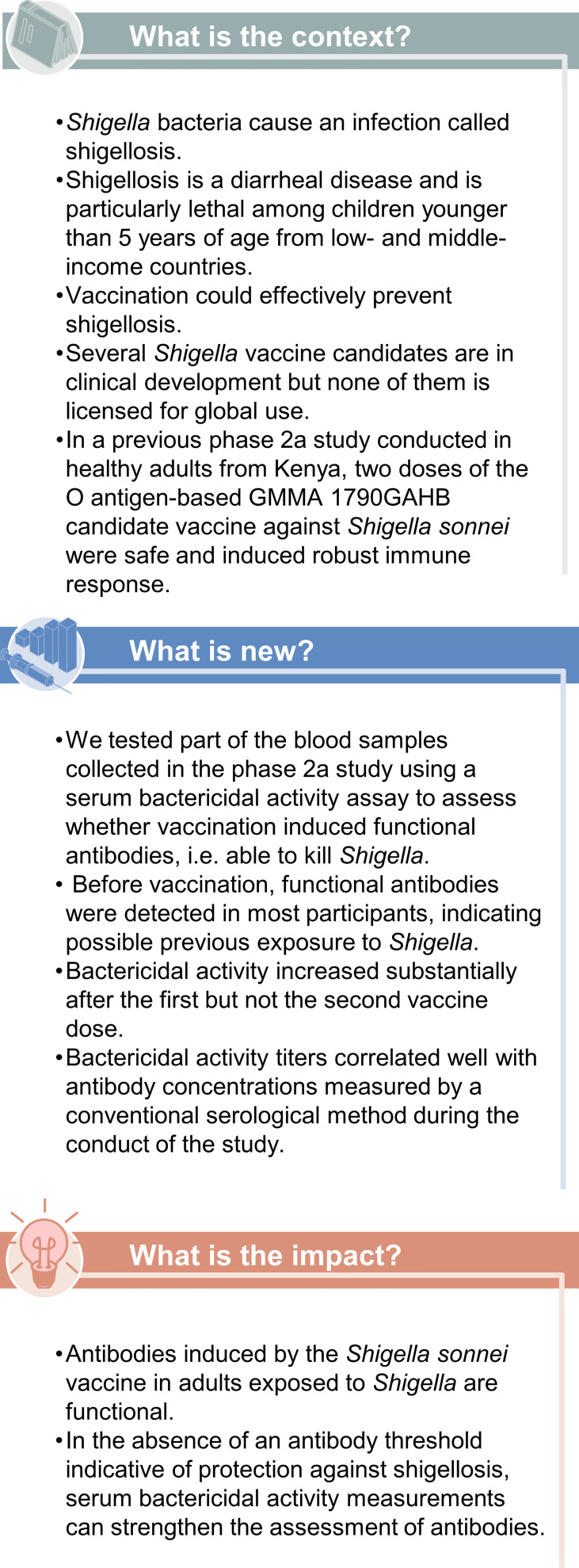
Plain language summary.

Here, we aimed to assess the functionality of antibodies induced by *S. sonnei* 1790GAHB GMMA in sera obtained from a phase 2 clinical trial conducted in Kenyan adults with known background immunity, including increased antibody levels to *S. sonnei* LPS OAg. A summary contextualizing the results and potential clinical relevance and impact of the research is provided in the Plain Language Summary (**Figure 1**).

## Materials and methods

### Study design and participants

In this *post-hoc* analysis, archived serum samples from a phase 2a, observer-blind, randomized, single-center, controlled study were used. The study was conducted at the Kenya Medical Research Institute (KEMRI)-Wellcome Trust in Kilifi, Kenya, between August 2016 and March 2017 (NCT02676895), as previously described (27). In brief, healthy adults aged 18–45 years were enrolled and randomized in a 1:1:1 ratio to receive 2 vaccinations with the investigational 1790GAHB vaccine (GSK) formulation containing 1.5 µg OAg/25 µg total protein (hereafter referred to as the 1.5/25 µg group), or 1790GAHB vaccine formulation containing 6 µg OAg/100 µg total protein (6/100 µg group), or control vaccines (Control group) at day (D) 1 and D29. As GMMA contain both OAg and protein components, their quantification is provided both in terms of OAg and protein amount (35–37). Participants in the control group received a quadrivalent meningococcal conjugate vaccine (*Menveo*, GSK) at D1 and a vaccine against tetanus, diphtheria, and acellular pertussis (*Boostrix*, GSK) at D29. Further details on study design and participants and full description of inclusion/exclusion criteria are disclosed in the primary publication (27). The trial was designed and conducted in accordance with the Good Clinical Practice Guidelines and the Declaration of Helsinki. Written informed consent was obtained before enrolment from each participant.

The current analysis evaluating SBA against *S. sonnei* at pre-vaccination (D1) and 28 days after the first (D29) and second dose (D57) of 1790GAHB vaccine was approved by the KEMRI Science and Ethics Review Unit (KEMRI/SERU/CGMR-C/179/3952) and the University of Oxford Tropical Ethics Committee (OxTREC 559-19).

### Procedures

The SBA analysis used only samples obtained from volunteers who agreed, as part of the informed consenting process, to the reuse of stored serum for further research, who received both vaccinations, and who provided blood samples at all time points. Blood samples were kept frozen at −80°C for further analysis. Not all available serum samples were assessed; exclusion criteria were budget constraints, insufficient volume of serum for testing, and samples with clinically implausible results, as assessed during the primary immunogenicity analysis (based on enzyme-linked immunosorbent assay [ELISA]) (27).

The bactericidal activity of antibodies induced by 1790GAHB against *S. sonnei* 53G *virG::cat* was assessed using the high-throughput luminescence-based SBA assay, developed at the GSK Vaccines Institute for Global Health (38). Detailed description of the assay has recently been published (34). Briefly, different dilutions of heat-inactivated test sera in phosphate buffer saline (PBS) at pH 7 in the presence of exogenous complement (baby rabbit complement) and bacteria were incubated. The heat-inactivated sera were serially diluted in PBS in the SBA plate. Log-phase cultures (optical density [OD] 600 = 0.22 ± 0.02) were prepared by starting a fresh inoculum at OD600 = 0.05 at 37°C (stirring at 180 rpm from an overnight liquid culture) and diluted to approximately 1 × 10^6^ colony forming unit/mL in PBS when log-phase was reached. The reaction mixture containing the target bacterial cells (20,000–25,000 bacteria per well), baby rabbit complement (at 20% final concentration), and PBS medium was added to each well of the SBA plate containing heat-inactivated serum dilutions, and the plates were incubated for 3 h at 37°C. After centrifugation, the supernatant was discarded and the remaining live bacterial pellets were resuspended in PBS, transferred in a white round-bottom 96-well plate and mixed (1:1 volumes) with BacTiter-Glo Reagent (Promega, Southampton, United Kingdom). The mixture was incubated for 5 minutes at room temperature on an orbital shaker, and the luminescence signal was measured by a luminometer (Synergy HT, Biotek, Swindon, UK).

The lower limit of quantification (LLOQ) was set to 33.5 inhibitory concentration (IC)50, while half the LLOQ (16.75) was assigned to participants with antibody titers below the LLOQ for the purpose of statistical analysis. IC50 represented the reciprocal of the serum dilution that resulted in killing 50% of the bacteria present in the assay.

### Statistical analysis

This was an exploratory analysis; therefore, the sample size calculation was not driven by statistical considerations but was based on samples available for testing. SBA geometric mean titers (GMTs) and associated 95% confidence intervals (CIs) were calculated at each time point by exponentiating (base 10) the corresponding arithmetic means and the lower and upper limit of the 95% CIs of the arithmetic means of logarithmically-transformed titers (base 10). Geometric mean ratios (GMRs) and associated 95% CIs were calculated for titers at D29 and D57 versus D1 by exponentiating (base 10) the mean within-participant differences in logarithmically-transformed titers and the corresponding 95% CIs. The number and percentage of participants with a 4-fold increase in SBA titers at D29 and D57 as compared to D1 were also computed with their 95% Clopper-Pearson CIs.

Description of anti-*S. sonnei* LPS serum IgG antibody responses was reported in the primary publication (27). The correlation between SBA titers and anti-*S. sonnei* LPS serum IgG antibody concentrations was assessed by calculating Pearson’s correlation coefficient *r* on logarithmically-transformed (base 10) values at all time points and overall.

## Results

In total, sera from 65 consenting participants met inclusion criteria. Ten participants from the Control group were excluded due to budget constraints and 5 participants were excluded due to clinically implausible results, as previously described in detail (27). Thus, 150 serum samples from 50 participants were included in the current analysis: 19 participants from the 1.5/25 µg group; 22 participants from the 6/100 µg group; and 9 participants from the Control group. The mean age at first vaccination was 26.9 years and 90% of participants were male (**Table 1**).

**Table 1:** Demographic characteristics of the study participants included in the SBA analysis.

	1.5/25 µg group (N=19)	6/100 µg group (N=22)	Control group (N=9)	Total (N=50)
Age (mean ± SD), years	25.5 ± 6.2	27.1 ± 8.0	29.4 ± 6.9	26.9 ± 7.1
Male, n (%)	18 (94.7)	19 (86.4)	8 (88.9)	45 (90.0)
Female, n (%)	1 (5.3)	3 (13.6)	1 (11.1)	5 (10.0)
Race, n (%)				
Black	18 (94.7)	22 (100.0)	9 (100.0)	49 (98.0)
White	1 (5.3)	0 (0.0)	0 (0.0)	1 (2.0)

SBA, serum bactericidal activity; N, total number of participants; n (%), number (percentage) of participants in each category; SD, standard deviation.;1.5/25 µg group, participants receiving the 1.5/25 µg O antigen/protein vaccine formulation; 6/100 µg group, participants receiving the 6/100 µg O antigen/protein vaccine formulation; Control, participants receiving a quadrivalent meningococcal conjugate vaccine at Day 1 and a vaccine against tetanus, diphtheria, and acellular pertussis at Day 29.

**Figure 2:**
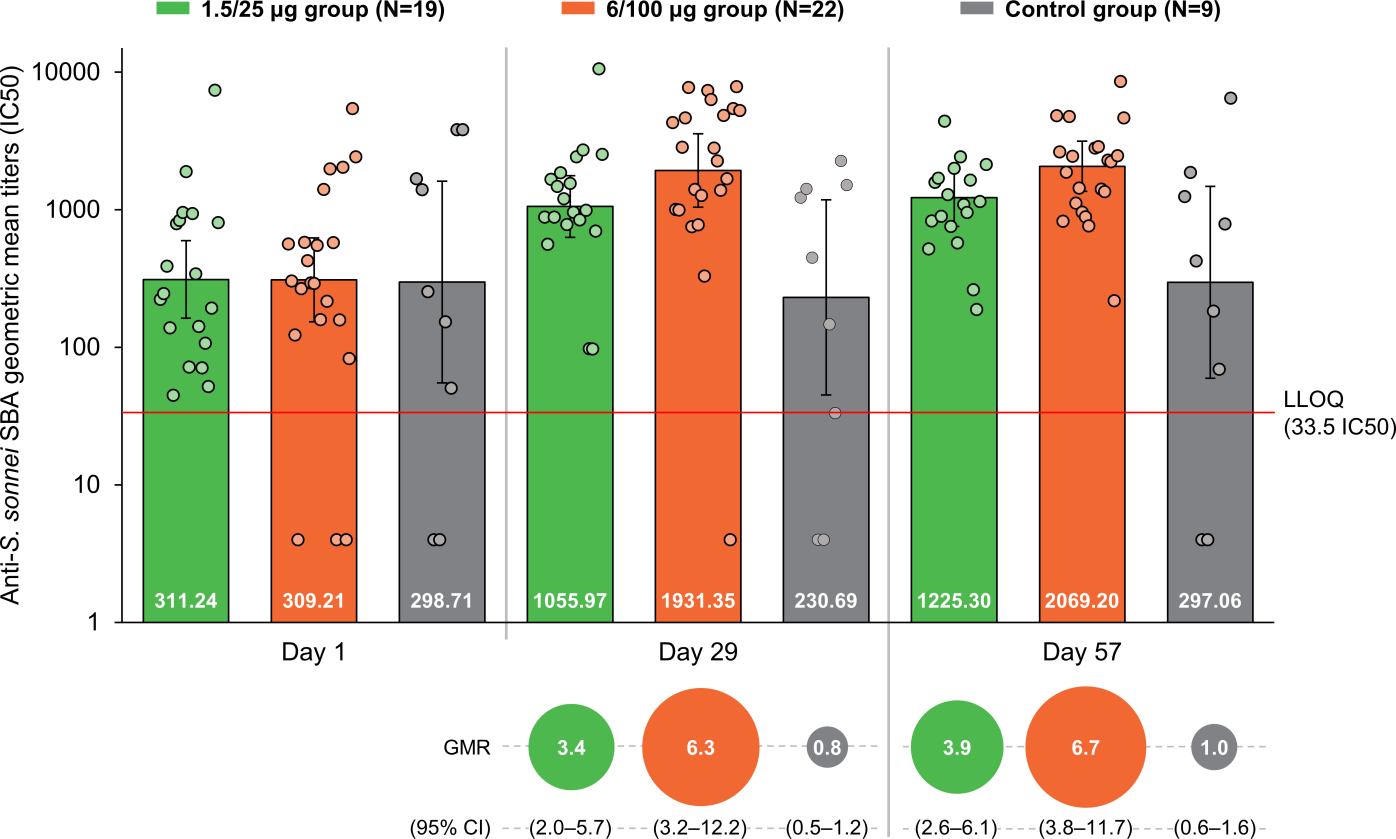
SBA geometric mean titers and within-participant geometric mean ratios at all time points (full analysis set). SBA, serum bactericidal activity; GMR, geometric mean ratio; CI, confidence interval; IC50, reciprocal of the serum dilution that results in killing 50% of the bacteria present in the assay; LLOQ, the lower limit of quantification of the assay; Day 1, pre-vaccination; Day 29, 28 days post-first vaccination; Day 57, 28 days post-second vaccination; 1.5/25 µg group, participants receiving the 1.5/25 µg O antigen/protein vaccine formulation; 6/100 µg group, participants receiving the 6/100 µg O antigen/protein vaccine formulation; Control, participants receiving a quadrivalent meningococcal conjugate vaccine at Day 1 and a vaccine against tetanus, diphtheria, and acellular pertussis at Day 29. Points represent individual data. Numbers at the base of the bars are the GMT values. Error bars depict 95% CIs. Note: Half the LLOQ (16.75 IC50) was assigned to participants with antibody titers below the LLOQ when calculating SBA GMTs.

**Figure 3:**
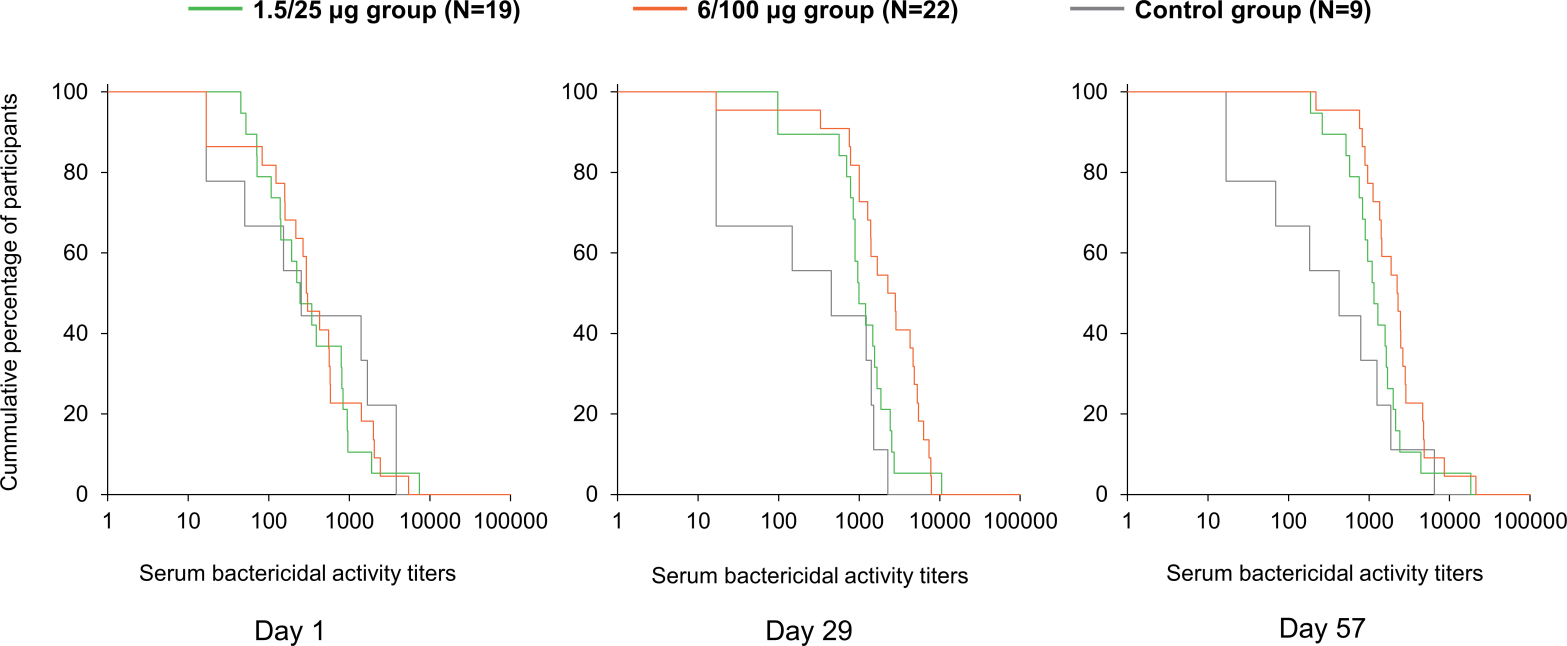
Reverse cumulative distribution curves for SBA titers (full analysis set). Day 1, pre-vaccination; Day 29, 28 days post-first vaccination; Day 57, 28 days post-second vaccination; 1.5/25 µg group, participants receiving the 1.5/25 µg O antigen/protein vaccine formulation; 6/100 µg group, participants receiving the 6/100 µg O antigen/protein vaccine formulation; Control, participants receiving a quadrivalent meningococcal conjugate vaccine at Day 1 and a vaccine against tetanus, diphtheria, and acellular pertussis at Day 29.

The baseline (D1) SBA titers were above the LLOQ in 90% (45/50) of participants. SBA GMTs were 311.2 (95% CI: 162.5–596.1) in the 1.5/25 µg group, 309.2 (95% CI: 153.4–623.4) in the 6/100 µg group and 298.7 (95% CI: 55.2–1616.8) in the Control group (**Figure 2**). At D29, SBA GMTs increased to 1056.0 (95% CI: 630.4–1768.7) and 1931.4 (95% CI: 1044.3–3572.0) in the 1.5/25 µg and 6/100 µg group, respectively. A slight increase was further observed at D57. In the Control group, GMT levels remained similar to those observed at D1 at both post-vaccination time points. When comparing post-vaccination GMTs to pre-vaccination titers, GMRs were ≥3.4 in the 1.5/25 µg group and ≥6.3 in the 6/100 µg group (**Figure 2**). In the 1.5/25 µg group, 32% (6/19) of participants and 42% (8/19) of participants reached 4-fold increase in SBA titers at D29 and D57, respectively. In the 6/100 µg group, 4-fold increases were detected in 50% (11/22) of participants at D29 and 64% (14/22) of participants at D57. Reverse cumulative distribution curves also indicated an elevated SBA by D29 as compared to D1 (**Figure 3**).

**Figure 4:**
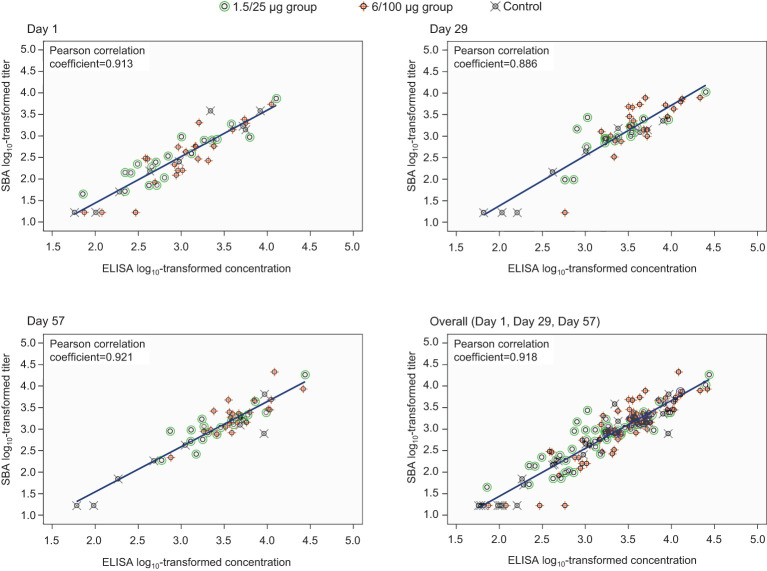
Correlation between anti-*S. sonnei* LPS serum IgG antibody concentrations and SBA titers (full analysis set). SBA, serum bactericidal activity; ELISA, enzyme-linked immunosorbent assay; IgG, immunoglobulin G; Day 1, pre-vaccination; Day 29, 28 days post-first vaccination; Day 57, 28 days post-second vaccination; 1.5/25 µg group, participants receiving the 1.5/25 µg O antigen/protein vaccine formulation; 6/100 µg group, participants receiving the 6/100 µg O antigen/protein vaccine formulation; Control, participants receiving a quadrivalent meningococcal conjugate vaccine at Day 1 and a vaccine against tetanus, diphtheria, and acellular pertussis at Day 29.

A strong positive correlation between SBA titers and anti-*S. sonnei* LPS IgG antibody concentrations was observed, the Pearson coefficients being ≥0.886 across all timepoints and 0.918 overall (**Figure 4**). Individual data are provided in **Supplementary Table 1**.

## Discussion

This study is the first to present SBA against *S. sonnei* in African, shigellosis-endemic settings, providing initial evidence that SBA is augmented by vaccination with 1790GAHB. A certain level of background exposure to *Shigella* is expected in endemic settings even among children under 5 years of age, who are the age group at the highest risk and should be primarily targeted by vaccination. An effective vaccine would therefore need to improve the bactericidal activity present at baseline. Most of the participants in this study were likely to be previously exposed to *Shigella* as confirmed by the detectable pre-vaccination titers. Pre-vaccination SBA GMTs (≥298.7) were slightly higher than levels observed after 2 doses of 1790GAHB, in individuals who participated in a phase 2b human challenge trial from the United States (US) (171.1 after receiving the 1.5/25 µg formulation) (39) or in the phase 1 study from France (237 after the 6/100 µg formulation in participants with baseline anti-*S. sonnei* LPS serum IgG antibody levels above or equal to the LLOQ for ELISA) (34). Despite these high background titers in African adults, 1790GAHB was able to further increase the levels of functional antibodies; 28 days after the first dose, titers were 3.4-fold higher in the 1.5/25 µg group and 6.3-fold higher in the 6/100 µg group as compared to D1, indicating a clear association between the OAg dose in the vaccine formulation and bactericidal antibody levels induced by the vaccine. SBA GMTs were elevated post-first immunization, and only increased slightly at D57. The same trend was previously observed for the anti-*S. sonnei* LPS serum IgG antibody concentrations measured by ELISA during the primary study. At D29, compared to baseline, antibody levels increased 2.1-fold (from 971 to 2038) in the 1.5/25 µg group and 4.4-fold (from 1196 to 5301) in the 6/100 µg group, while no substantial increase was observed following the second dose (27). Taken together, the SBA results of this analysis and IgG antibody responses assessed in all participants in the primary study (27) suggest that an increased OAg/protein dose might enhance the immunogenicity of future *S. sonnei* GMMA vaccine formulations. We observed a strong correlation between SBA titers and anti-*S. sonnei* LPS serum IgG antibody concentrations at all time points, as previously reported post-vaccination with 1790GAHB (34).

Trends in the SBA levels over time in the present analysis were in line with those observed in participants from countries non-endemic to shigellosis. In the phase 1 dose-escalation study from France, SBA levels increased 2.9-fold from D1 to D29 in participants who received the 1.5/25 µg dose and 3.8-fold in those receiving the 6/100 µg dose. The study also demonstrated the persistence of functional antibody levels up to 6 months after vaccination (34). In the *S. sonnei* human challenge study in the US, all participants received the 1.5/25 µg vaccine formulation. Those individuals who did not develop shigellosis following the administration of the challenge agent, had significantly higher SBA GMTs at both D1 compared with individuals who developed shigellosis post-challenge; GMRs (D29 *versus* D1) were also higher in protected (3.9) than unprotected (1.5) individuals (39). These results suggest a protective effect of high bactericidal antibody levels against shigellosis, which is paramount, particularly in endemic settings. Literature data related to the functionality of antibodies induced by *Shigella* vaccines are scarce. A recent study also reported a strong association between *S. flexneri* 2a-specific SBA titers in human adult volunteers and reduced clinical disease post-challenge with wild-type organisms (40), thus supporting the value of SBA assays to potentially predict vaccine efficacy. SBA has already been assessed during the clinical development of several vaccines against *Shigella*. In a study conducted by Cohen et al. in healthy adult volunteers from Israel, robust post-vaccination SBA titers were reported for the synthetic carbohydrate-based conjugate vaccine SF2a-TT15 against *S. flexneri* as compared to baseline (18). In a human challenge study conducted in the US, following vaccination with Flexyn2a (containing the *S. flexneri* 2a O-polysaccharide to *Pseudomonas aeruginosa* exotoxin), vaccinees protected had higher SBA titers compared to those not protected post-challenge (41). In another study conducted among Bangladeshi adults and children, the live-attenuated oral candidate vaccine against *S. sonnei* (WRSS1) did not induce SBA in adults as compared to placebo recipients, but a significant increase in SBA titers was detected in children who received WRSS1 (42).

The percentage of participants with 4-fold increase in post-vaccination SBA titers, as compared to titers at D1, tended to increase with OAg/protein dose, reaching 42% in the 1.5/25 µg group and 64% in the 6/100 µg group at D57. However, these data should be carefully interpreted. The high pre-vaccination SBA titers observed in this study may have resulted in some participants not reaching 4-fold increase, despite achieving high SBA levels. The use of a 4-fold criterion might lead to discordant results as it can underestimate the number of responders to vaccination in populations with high pre-existing antibody levels (43). Moreover, there is no evidence that the observed 4-fold increase in IgG antibody levels can be directly correlated with fold-increases in SBA titers because not all IgG subclasses fix complement equally (44, 45) and other immunoglobulin isotypes with complement-fixing activities are also present in the serum.

The small sample size might be considered as a potential limitation of the study. Additionally, 89% of participants in the primary study, and as a consequence most participants (90%) included in our analysis, were male which may limit the generalizability of the results. However, there is no literature indicating any differences between Kenyan males and females in terms of immune response, frequency of vaccination-related adverse events, or susceptibility to infection with *Shigella*.

The high-throughput luminescence-based SBA assay used in this study is a well-established method, able to analyze a large number of samples in a short period of time, requiring small volumes of serum and providing consistent results as demonstrated in previous studies (34, 39). Therefore, it can be suitably used in future trials to assess the functionality of antibodies induced by *Shigella* vaccines. Moreover, the optimization of the luminescence-based SBA, by reduction in the LLOQ from 100 used in previous trials to 33.5 applied here (38), allowed higher sensitivity analysis of functional antibodies and a more accurate characterization of immune response to 1790GAHB in an adult population with pre-existing antibodies to *S. sonnei*.

In conclusion, vaccination with 1790GAHB increased baseline SBA levels in adults from shigellosis-endemic settings. SBA titers strongly correlated with anti-*S. sonnei* LPS serum IgG antibody concentrations at all timepoints, indicating robust bactericidal activity of antibodies triggered by both natural exposure and vaccination. The mechanisms of protection against *Shigella* are multiple and have not fully been elucidated. However, antibody functionality, including bactericidal activity and opsonophagocytosis, may also be correlated with protective immunity against shigellosis (33, 40). Therefore, when evaluating immune responses, both the functionality and the binding capacity of vaccine-induced *Shigella*-specific antibodies should be assessed. There is no established correlate of protection against shigellosis for either ELISA or SBA, though considerable effort is devoted for standardizing ELISA measurements and ensuring comparability across different vaccine development programs. In the absence of a protective ELISA threshold and considering the strong correlation between the assays, SBA can effectively complement ELISA data by indicating the functionality of vaccine-induced antibodies.

## Data availability statement

The datasets presented in this study can be found in online repositories. The names of the repository/repositories and accession number(s) can be found below: Anonymized individual participant data and study documents can be provided upon request from www.clinicalstudydatarequest.com.

## Ethics statement

The current analysis evaluating SBA against S. sonnei at pre-vaccination (D1) and 28 days after the first (D29) and second dose (D57) of 1790GAHB vaccine was approved by the KEMRI Science and Ethics Review Unit (KEMRI/SERU/CGMR-C/179/3952) and the University of Oxford Tropical Ethics Committee (OxTREC 559-19). The patients/participants provided their written informed consent to participate in this study.

## Author contributions

AA, PB, VC, LM, FM, UN, FN, PN, CO, AP, OR, ES and AS were involved in the study conception and design. AA, MK, LM, UN, CO, AP, OR and ES were involved in acquisition and generation of data. MK, LM, UN, FN, OR and ES performed the study. AA, LM, FM, FN, CO, OR and ES contributed to materials/analysis/reagent tools. PB, VC, MK, FM, UN, FN, PN, AP, OR and ES were involved in data analysis and data interpretation. All authors contributed substantially to the development of the manuscript and approved the final version.

## Funding

GlaxoSmithKline Biologicals SA funded this analysis and took responsibility for all costs associated with the development of the present manuscript.

## Acknowledgments

The contribution of study participants and the current and former GVGH staff members is gratefully acknowledged, and especially Antonio Di Pasquale, Elisa Marchetti, Joachim Auerbach, Augustin G. W. Ndiaye. The authors thank Kishor Mariyala, Sateesh Aravapalli, and Rob Mulder for providing statistical programming activities. The authors would also like to thank Modis for editorial assistance, manuscript coordination, and design support, on behalf of GSK. Botond Nagy and Petronela Petrar provided medical writing support; Gil Costa provided design support, and Ana de la Grandiere coordinated manuscript development.

## Conflict of interest

The study was funded by GlaxoSmithKline Biologicals SA. The funder was involved in study design, data collection and analysis, decision to publish and preparation of the manuscript. AA, VC, FM UN, FN, OR, ES and AS are employees of the GSK group of companies. LM and AP were employees of the GSK group of companies at the time of the study and are no longer affiliated with GSK. VC, LM and AP hold shares in the GSK group of companies. PB institution received funding from GSK group of companies for the conduct of the present study. Laura B. Martin declares 2 patents (WO2016202872 – Immunogenic compositions & WO2021074352 – Novel vaccine compositions).

The remaining authors declare that the research was conducted in the absence of any commercial or financial relationships that could be constructed as a potential conflict of interest.

## Publisher’s note

All claims expressed in this article are solely those of the authors and do not necessarily represent those of their affiliated organizations, or those of the publisher, the editors and the reviewers. Any product that may be evaluated in this article, or claim that may be made by its manufacturer, is not guaranteed or endorsed by the publisher.
