# Supplementary material for: Complement-mediated serum bactericidal activity of antibodies elicited by the Shigella sonnei GMMA vaccine in adults from a shigellosis-endemic country: Exploratory analysis of a Phase 2a randomized study
Source: Front Immunol. 2022 Sep 9;13:971866. doi: 10.3389/fimmu.2022.971866 (PMC9531247; doi:10.3389/fimmu.2022.971866)
Supplement: Supplementary file 1 [file Table_1.docx]

## Supplementary Table 1. Individual data for SBA against *S. sonnei* 53G *virG::cat* as assessed through a high-throughput luminescence-based SBA assay and anti-*S. sonnei* LPS IgG concentration assessed with ELISA, at each timepoint

| 1.5/25 µg group | | |  | 6/100 µg group | | |  | Control group | | |
| --- | --- | --- | --- | --- | --- | --- | --- | --- | --- | --- |
| Participant number | SBA titer (IC50) | Anti-*S. sonnei* LPS IgG conc (EU/mL) |  | Participant number | SBA titer (IC50) | Anti-*S. sonnei* LPS IgG conc (EU/mL) |  | Participant number | SBA titer (IC50) | Anti-*S. sonnei* LPS IgG conc (EU/mL) |
| Day 1 |  |  |  |  |  |  |  |  |  |  |
| 1 | 222.8 | 310.6 |  | 1 | 565.2 | 1481.5 |  | 1 | 1680.7 | 5212.5 |
| 2 | 245.5 | 505.3 |  | 2 | 303.1 | 383.9 |  | 2 | 1398.0 | 5574.1 |
| 3 | 388.6 | 1314.7 |  | 3 | 123.2 | 875.1 |  | 3 | 253.7 | 944.9 |
| 4 | 138.5 | 259.4 |  | 4 | 4.0 | 74.7 |  | 4 | 4.0 | 101.8 |
| 5 | 44.8 | 71.9 |  | 5 | 266.7 | 2053.2 |  | 5 | 4.0 | 57.4 |
| 6 | 791.2 | 1843.7 |  | 6 | 580.1 | 2393.8 |  | 6 | 153.0 | 431.7 |
| 7 | 835.5 | 2603.4 |  | 7 | 426.3 | 1129.0 |  | 7 | 50.5 | 191.6 |
| 8 | 955.2 | 1003.9 |  | 8 | 294.1 | 1562.3 |  | 8 | 3829.6 | 8268.7 |
| 9 | 1896.7 | 3831.2 |  | 9 | 291.8 | 406.3 |  | 9 | 3826.5 | 2189.0 |
| 10 | 71.8 | 521.1 |  | 10 | 552.6 | 919.8 |  |  |  |  |
| 11 | 940.4 | 6227.0 |  | 11 | 158.8 | 1031.9 |  |  |  |  |
| 12 | 341.8 | 701.4 |  | 12 | 1408.4 | 3960.1 |  |  |  |  |
| 13 | 141.5 | 222.8 |  | 13 | 216.6 | 836.0 |  |  |  |  |
| 14 | 70.8 | 421.2 |  | 14 | 1983.0 | 5600.0 |  |  |  |  |
| 15 | 107 | 639.0 |  | 15 | 577.4 | 1457.1 |  |  |  |  |
| 16 | 52 | 221.7 |  | 16 | 4.0 | 294.8 |  |  |  |  |
| 17 | 192.6 | 456.7 |  | 17 | 158.1 | 915.4 |  |  |  |  |
| 18 | 7414.8 | 12681.3 |  | 18 | 2042.7 | 1604.8 |  |  |  |  |
| 19 | 805.8 | 2282.1 |  | 19 | 4.0 | 119.6 |  |  |  |  |
|  |  |  |  | 20 | 83.0 | 493.5 |  |  |  |  |
|  |  |  |  | 21 | 5447.7 | 11182.3 |  |  |  |  |
|  |  |  |  | 22 | 2428.8 | 5432.2 |  |  |  |  |
| D29 |  |  |  |  |  |  |  |  |  |  |
| 1 | 886.0 | 1680.7 |  | 1 | 4298.8 | 10624.8 |  | 1 | 1226.8 | 4308.5 |
| 2 | 562.3 | 1035.4 |  | 2 | 1003.8 | 1962.1 |  | 2 | 1417.6 | 5250.3 |
| 3 | 1667.0 | 3317.5 |  | 3 | 998.6 | 5224.4 |  | 3 | 448.9 | 1013.6 |
| 4 | 885.7 | 1722.2 |  | 4 | 2855.3 | 3248.3 |  | 4 | 4.0 | 107.5 |
| 5 | 1478.7 | 798.1 |  | 5 | 4653.1 | 3509.7 |  | 5 | 4.0 | 65.6 |
| 6 | 1856.1 | 3518.7 |  | 6 | 7752.5 | 4967.7 |  | 6 | 146.9 | 414.5 |
| 7 | 1203.9 | 3465.9 |  | 7 | 754.9 | 2594.8 |  | 7 | 33.2 | 161.0 |
| 8 | 781.1 | 2194.9 |  | 8 | 1405.1 | 4742.0 |  | 8 | 2266.4 | 7958.3 |
| 9 | 1556.9 | 3980.2 |  | 9 | 778.7 | 2226.9 |  | 9 | 1515.1 | 2394.4 |
| 10 | 962.4 | 2367.6 |  | 10 | 1273.8 | 1527.6 |  |  |  |  |
| 11 | 2429.5 | 9210.5 |  | 11 | 330.1 | 2157.5 |  |  |  |  |
| 12 | 846.3 | 1759.5 |  | 12 | 7359.9 | 13286.8 |  |  |  |  |
| 13 | 2724.6 | 1057.7 |  | 13 | 6319.4 | 12803.7 |  |  |  |  |
| 14 | 990.9 | 3364.7 |  | 14 | 2815.2 | 8982.3 |  |  |  |  |
| 15 | 97.7 | 730.7 |  | 15 | 2264.6 | 3611.2 |  |  |  |  |
| 16 | 97.3 | 578.4 |  | 16 | 1384.5 | 5142.5 |  |  |  |  |
| 17 | 699.4 | 1683.3 |  | 17 | 4842.6 | 3173.8 |  |  |  |  |
| 18 | 10597.9 | 25043.0 |  | 18 | 1682.8 | 3230.7 |  |  |  |  |
| 19 | 2529.4 | 4738.6 |  | 19 | 4.0 | 578.6 |  |  |  |  |
|  |  |  |  | 20 | 5442.3 | 4277.2 |  |  |  |  |
|  |  |  |  | 21 | 7864.0 | 21501.3 |  |  |  |  |
|  |  |  |  | 22 | 5261.8 | 8533.9 |  |  |  |  |
| Day 57 |  |  |  |  |  |  |  |  |  |  |
| 1 | 519.8 | 1288.2 |  | 1 | 4829.1 | 11056.5 |  | 1 | 1250.4 | 4813.1 |
| 2 | 828.5 | 2018.3 |  | 2 | 2635.5 | 2386.7 |  | 2 | 1864.4 | 4862.1 |
| 3 | 1586.4 | 3844.1 |  | 3 | 824.2 | 3867.6 |  | 3 | 424.2 | 1109.4 |
| 4 | 1698.8 | 1735.4 |  | 4 | 1880.1 | 3762.2 |  | 4 | 4.0 | 96.1 |
| 5 | 895.2 | 743.5 |  | 5 | 4758.2 | 3518.9 |  | 5 | 4.0 | 61.0 |
| 6 | 4409.3 | 7214.6 |  | 6 | 2453.8 | 6230.1 |  | 6 | 183.6 | 480.8 |
| 7 | 1287.0 | 4053.3 |  | 7 | 1120.1 | 3272.9 |  | 7 | 69.4 | 183.8 |
| 8 | 758.4 | 2590.5 |  | 8 | 1441.1 | 5722.7 |  | 8 | 788.2 | 9147.4 |
| 9 | 2003.5 | 4804.7 |  | 9 | 959.7 | 2207.6 |  | 9 | 6464.6 | 9301.3 |
| 10 | 574.1 | 1777.0 |  | 10 | 887.1 | 1826.1 |  |  |  |  |
| 11 | 2422.7 | 9685.9 |  | 11 | 764.9 | 2579.8 |  |  |  |  |
| 12 | 1090.0 | 2868.9 |  | 12 | 21307.5 | 12095.8 |  |  |  |  |
| 13 | 957.9 | 1300.1 |  | 13 | 2806.3 | 10699.5 |  |  |  |  |
| 14 | 1639.9 | 5220.9 |  | 14 | 2876.0 | 10232.7 |  |  |  |  |
| 15 | 262.1 | 1491.8 |  | 15 | 1412.2 | 4491.8 |  |  |  |  |
| 16 | 188.6 | 592.0 |  | 16 | 1356.1 | 3641.6 |  |  |  |  |
| 17 | 1149.2 | 1837.9 |  | 17 | 2296.3 | 4651.7 |  |  |  |  |
| 18 | 18367.3 | 27449.2 |  | 18 | 2239.6 | 3949.0 |  |  |  |  |
| 19 | 2133.5 | 5950.2 |  | 19 | 218.7 | 756.3 |  |  |  |  |
|  |  |  |  | 20 | 2473.7 | 3623.3 |  |  |  |  |
|  |  |  |  | 21 | 8583.1 | 26140.7 |  |  |  |  |
|  |  |  |  | 22 | 4656.7 | 7082.3 |  |  |  |  |

SBA, serum bactericidal activity; LPS, lipopolysaccharide; IgG, immunoglobulin G; ELISA, enzyme-linked immunosorbent assay;1.5/25 µg group, participants receiving the 1.5/25 µg O antigen/protein vaccine formulation; 6/100 µg group, participants receiving the 6/100 µg O antigen/protein vaccine formulation; Control, participants receiving a quadrivalent meningococcal conjugate vaccine at Day 1 and a vaccine against tetanus, diphtheria, and acellular pertussis at Day 29; IC50, reciprocal of the serum dilution that results in killing 50% of the bacteria present in the assay; EU, ELISA units; Day 1, pre-vaccination; Day 29, 28 days post-first vaccination; Day 57, 28 days post-second vaccination.
